# Dietary Regimen, Overweight, and Obesity in Human Nutrition Students and Other Majors: A Cross-Sectional Study

**DOI:** 10.1155/2022/9957690

**Published:** 2022-03-23

**Authors:** Fernando D. Solís-Guevara, Percy G. Ruiz Mamani, Jacksaint Saintila

**Affiliations:** ^1^Escuela de Nutrición Humana, Universidad Peruana Unión, Lima, Peru; ^2^Sociedad Científica de Estudiantes de Nutrición de la Universidad Peruana Unión (SOCENUT-UPeU), Lima, Peru; ^3^Grupo de Investigación en Nutrición y Estilo de Vida (GINEV), Escuela de Nutrición Humana, Universidad Peruana Unión, Lima, Peru; ^4^Escuela Profesional de Enfermería, Facultad de Ciencias de la Salud, Universidad Privada San Juan Bautista, Lima, Peru

## Abstract

**Background:**

Choosing a healthy diet is an increasingly a challenge for university students. The objective of this study was to compare diet and overweight/obesity in human nutrition students (HNS) and students of other careers (SOC) from a university located in Lima, Peru.

**Methods:**

It was a cross-sectional study consisting of 158 students out of an initial sample of 170. Information was collected on the sociodemographic and anthropometric characteristics of the participants and a validated questionnaire was applied to evaluate the frequency of food consumption.

**Results:**

There was no significant difference in diet between HNS and SOC (*p* > 0.05). HNS most frequently consumed yellow/orange vegetables (*p* = 0.020), purple vegetables (*p* = 0.049), citrus fruits (*p* = 0.029), eggs (*p* = 0.002), whole milk (*p* = 0.013), yogurt (*p* = 0.017), tofu (*p* = 0.003), olive oil (*p* = 0.003), other vegetable oils (*p* < 0.001), and alcoholic beverages (*p* = 0.037) than SOC. In contrast, HNS had a lower frequency of nonfried tuber intake (*p* = 0.039), fried tubers (*p* < 0.001), milk desserts (*p* = 0.048), flour fritters (*p* = 0.027), cookies with chocolate (*p* = 0.050), croissants (*p* = 0.030), cookies with filling (*p* = 0.024), candies (*p* = 0.006), and soda (*p* = 0.016) than SOC. Overweight/obesity was not different between HNS and SOC (*p* > 0.05).

**Conclusions:**

This study found significant differences in the consumption of some foods between HNS and SOC. However, it found no significant difference in diet and overweight/obesity between HNS and SOC.

## 1. Introduction

University is a major change for university students in terms of the risk of excess body weight, especially during the first year of study [1]. Obesity represents one of the greatest challenges for health systems. In addition, it constitutes a morbidity and mortality risk factor with a high economic cost. The global prevalence of obesity has doubled since 1980, affecting one-third of the population [2]. According to data from the World Health Organization (WHO), in 2016, 39% of adults over the age of 18 were overweight, while 13% were considered obese [3]. Annual deaths due to obesity amount to approximately 2.8 million [4]. Obesity is a serious problem in Peru. According to a report by the National Health Institute (INS), 22.3% of the population over 15 years of age suffers from obesity [5], these figures position Peru as one of the countries with the highest prevalence of obesity in the region.

In recent years, there has been a growing interest in researching dietary regimen, which is defined as the habitual consumption of foods, food groups, or nutrients that may vary in quantity, frequency, and combination of these in the diet [6]. Dietary regimens may be influenced by culture, ethnicity, personal philosophy, and environmental factors such as food availability, purchasing capacity, preparation methods, food advertising, and state policies [7]. Healthy diets, particularly those based on the consumption of abundant, minimally processed plant foods, such as vegetarian diets, have an important role in the prevention of noncommunicable diseases such as cardiovascular diseases, some types of cancers, type 2 diabetes mellitus, and obesity [8, 9]. This could be justified by the fact that vegetarian diets are characterized by a high intake of vegetables, fruits, whole grains, legumes, nuts, and seeds [10]. These foods are rich in dietary fiber and bioactive elements, which can provide benefits for the prevention, control, and treatment of various diseases [11].

Excess body weight in university students may be due to several factors, among the most important are the lack of regular physical activity and an inadequate diet characterized by a high content of foods rich in saturated fats, salt and free sugars [12]. Obesity, according to WHO, is the result of a series of risky eating behaviors, including unhealthy eating practices that begin during adolescence and continue through university [13]. In fact, a high prevalence of overweight and obesity [12], which associated with a low intake of fresh foods such as fruits, vegetables, and whole-grain foods [14]. In Peru, 88.7% of the population over 18 years of age does not consume 400 g/day or 5 servings/day of fruits and vegetables [15], the amount recommended by the WHO [13].

While several investigations were conducted on university students in to assess nutritional status [16, 17] and eating habits [18, 19]. However, few studies have focused on comparing anthropometric indicators such as BMI and food consumption frequency in HNS and SOC. Given that, presumably, the HNS are more knowledgeable about nutrition, it is expected that this will be reflected in better eating habits, lifestyle, food choices, and nutrient profiling. Consequently, it is important to explore the pattern of food consumption in Peruvian HNS and SOC. For this reason, the objective of this research was to compare the dietary regimen and overweight/obesity in HNS and SOC of a private university in Lima, Peru.

## 2. Materials and Methods

### 2.1. Design, Type of Research, and Participants

A cross-sectional study was carried out on 170 students, selected by non-probabilistic convenience sampling, considering all careers of a private university, located in Lima, Peru. The recruitment was during the months of October 2019 to March 2020, through invitations to fill out an online survey that was shared through social networks (Facebook and WhatsApp) and e-mail. Inclusion criteria were [1] being a university student enrolled at the institution, [2] being >18 years old, and [3] signing the electronic informed consent. Excluded were those that did not meet the above criteria (*n* = 8), surveys that were inadequately completed or provided invalid (*n* = 1) and duplicate (*n* = 3) information. The final sample was a total of 158 university students.

### 2.2. Data Collection Instruments

For the collection of sociodemographic, academic, medical, anthropometric, and dietary data, an online survey was created in Google Form to be self-administered by each participant. The survey was divided into 5 sections:

The first part covered the description of the study (presentation of the investigators, title, objective, and relevance of the study) and the informed consent form was also presented as proof of voluntary participation. It was considered that if the person clicked on the “Yes” option, he/she agreed to be part of the research.

The second part collected sociodemographic data (age, sex, place of origin, and religion), academic data (faculty, career, and academic year), medical (including illness diagnosed by a physician and use of medications), and anthropometric (self-reported weight and height). The latter data were used to calculate body mass index (BMI) and, subsequently, to classify them as lean (BMI <18.5), normal (BMI 18.5 and < 25.0), overweight (BMI 25.0 and < 30.0), and obesity (BMI ≥30.0), as proposed by the WHO [20].

The third section asked for dietary data; for example, the use of nutritional supplements and self-defined types of diet (vegetarian and nonvegetarian). The vegetarian diet was classified as follows: lacto-ovo-vegetarian, lacto-vegetarian, ovo-vegetarian, pesco-vegetarian, and vegan [21].

Finally, the fourth and fifth sections were composed of a Food Frequency Questionnaire (FFQ). It is composed of 58 items, with a validity of 1 according to Aiken's V and a reliability of >0.7 according to Cronbach's alpha, used in a previous research [22]. The FFQ was divided into 11 food groups: bread, cereals, and grains; tubers; vegetables; fruits; meats; dairy and dairy products; soy and soy products; oils and fats; legumes; sweets and pastries; and beverages. Likewise, the frequency of consumption was recorded considering the following scales: 0 = 0 times/week, 1 = 0.25 times/week, 2 = 0.5 times/week, 3 = 1.5 times/week, 4 = 3.5 times/week, 5 = 5.5 times/week and 6 = 7 times/week [22]. This FFQ was used to corroborate and correctly assess the diet, classifying as omnivorous those who consume meat (red meat, fish, and poultry), dairy products, and eggs on a regular basis; lacto-ovo-vegetarian consumes dairy products and eggs but not meat; lacto-vegetarian consumes dairy products but not eggs and meat; ovo-vegetarian consumes eggs but not dairy products and meats; pesco-vegetarian consumes fish, dairy products and eggs but not red meat and poultry, and vegans do not consume meat, dairy products and eggs [21]. All types of vegetarian diets were grouped into “vegetarians” for ease of analysis.

### 2.3. Statistical Analysis

Finally, the data collected were recorded and organized in a Microsoft Excel matrix, version 2013. For the descriptive analysis of the sociodemographic, academic, medical, anthropometric, and dietary data, absolute and relative frequency tables and in some cases the mean and standard deviation were used. The analysis of the difference in food consumption between HNS and SOC was performed with the Mann-Whitney U test, because the variables did not have a normal distribution. The Chi-square test was used to analyze the association between the type of career and diet. All these analyses were performed with IBM SPSS statistical software, version 25.0. Values of *p* < 0.05 were considered statistically significant.

## 3. Results

Table 1 presents the characteristics of the students. The mean age was 22.2 ± 3.1 and 22.3 ± 4.1 years for HNS and SOC, respectively. Women accounted for 72.5% in HNS, whereas in SOC the highest proportion (51.7%) was male. In general, students came mainly from the coast (50.6%). Membership in the Seventh-day Adventist Church (SDA) was the most common (93.7%). 68.4% belong to the Faculty of Health Sciences, where the Human Nutrition career had the highest participation (43.7%). Almost the same proportion belonged to the 3rd year (29.7%) and 4th year (31%) of study. Only 19 students reported having any illness, of which 10 indicated medications and 8 reported using nutritional supplements.

In Table 2, it was found that there is a higher prevalence of overweight/obesity in males than in females (*p* < 0.05). In addition, it was observed that the proportion of overweight/obesity did not differ significantly between ENH and EOC (*p* > 0.05). On the other hand, there was no difference in the proportion of self-reported vegetarians between ENH and EOC (*p* > 0.05). Finally, the proportion of overweight/obesity in self-reported vegetarian and nonvegetarian students did not differ significantly (*p* > 0.05).

Table 3 shows that the HNS most frequently consumed yellow/orange vegetables (carrot, pumpkin, and squash), purple vegetables (beet, eggplant, and purple cabbage), citrus fruits (orange, tangerine, kiwi, and strawberry), eggs, whole milk, yogurt, tofu, olive oil, and other vegetable oils (sunflower, corn or soybean oil), compared to SOC; there was a significant difference in these results (*p* < 0.05). In contrast, the HNS had a lower frequency of intake of nonfried tubers (potato, sweet potato, yucca, olluco, and chuño), fried tubers (fried potato or fried sweet potato), milk-based desserts (custard, pudding, and flan), flour fritters, chocolate cookies, croissants, cookies with fillings, candies (caramels and mints), and soft drinks than SOC, with statistically significant differences (*p* < 0.05). However, it was also observed that HNS consumed alcoholic beverages more frequently compared to SOC (*p* = 0.037).

In general, both groups of students presented a low frequency of consumption of foods such as meats (except for eggs, chicken, and fish); dairy products and derivatives; soybeans and derivatives; oils and fats (except avocado); sweets and pastries (except for blonde sugar or white sugar); and beverages (except pure water). While almost a medium-high frequency of bread consumption, cereals and grains; tubers (except for potato chips or fried sweet potatoes); vegetables; fruits (except for dehydrated fruits); and legumes, without presenting significant differences.

A higher percentage of HNS were vegetarians (18.8%). In contrast, the majority of SOC were nonvegetarians (93.3%). The result of the relationship between the type of career and dietary regimen evaluated was statistically significant (*p* = 0.020), as shown in Table 4.

## 4. Discussion

Diets based primarily on plant-based foods such as fruits, vegetables, whole grains, legumes, nuts, and seeds have been recognized as beneficial in preventing and treating various noncommunicable diseases, including overweight/obesity [11].

### 4.1. Comparison of Overweight/Obesity according to Type of Career and Self-Reported Diet

Obesity represents one of the biggest challenges for college students. University students are at greater risk of gaining weight than those who do not attend a university [23]. In the current study, the findings indicated that there was no statistically significant difference in overweight/obesity between HNS and SOC. These results are consistent with the findings of a study in which no differences in BMI were found between HNS and other careers [24]. These results are supported by the findings of a study comparing BMI values in human nutrition students and other careers, which found that there was no significant difference, evidencing that both groups presented BMI within normal ranges [25]. However, in another study, it was found that 90% of human nutrition students had a normal BMI, furthermore, when comparing the mean BMI, it was observed that HNS presented a lower value compared to another discipline [26]. Moreover, a study comparing the BMI of students majoring in nutrition and those not majoring in nutrition found that BMI was significantly different in both cohorts, reporting that human nutrition students had lower BMI, however, the BMI values for both groups were within normal ranges [27]. However, in our study, the proportion of human nutrition students who presented excess body weight was higher compared to those studying other university courses.

Our study also found no significant difference in overweight/obesity in students with vegetarian and nonvegetarian self-reported diets. These results are similar to those found by Bowman [28], where significant differences were found between vegetarians and nonvegetarians in overweight/obesity. On the other hand, Lee and Krawinkel [29], reported that Buddhist vegetarians had a high tendency toward overweight/obesity (13.0%) compared to Catholic nonvegetarians (7.0%) in South Korea; which differs from our findings (25.0% vs. 29.4% in vegetarians and nonvegetarians, respectively). This could be because the self-reported vegetarian students in our study were misclassified and because of the sample size. In contrast, our findings are different from those reported by Chiu et al. [30], who found a significant differences in the proportion of overweight (BMI >27.0 kg/m^2^) between Taiwanese vegetarians and nonvegetarians (*p* < 0.0001). In addition, Matsumoto et al. [31], found significant difference in overweight (BMI >25.0 kg/m^2^), and obesity between lacto-ovo-vegetarians/vegans and Adventist Health Study-2 (AHS-2) Adventist nonvegetarians (*p* < 0.0001 and *p* < 0.001, respectively). Likewise, in a Brazilian study, there was a significant difference (*p* = 0.002) in overweight (BMI >24.9 kg/m^2^) between vegetarians and nonvegetarians [32]. Similarly, significant differences were found in Peruvian vegetarians and nonvegetarians in overweight/obesity (*p* < 0.01) [33]. These findings are in agreement with the reports of Parra-Fernández et al. [34] and Paslakis et al. [35], finding that Spanish and German vegetarians/vegans, respectively, had a lower BMI. Overall, the current scientific literature supports that vegetarian and vegan diets are significantly associated with lower BMI compared to omnivores [36].

### 4.2. Comparison of Food Consumption Frequency between HNS and SOC

It is expected that HNS will have a better knowledge of nutrition and food, and that this will be reflected in healthy food choices [37]. In this regard, our study found that HNS had a significantly higher frequency of consumption of healthy foods, such as yellow/orange vegetables, purple vegetables, citrus fruits, eggs, whole milk, yogurt, tofu, olive oil, and other vegetable oils, except for alcoholic beverages; and a significantly lower frequency of consumption of unhealthy foods, e.g. fried tubers, milk-based desserts, flour fried foods, chocolate cookies, croissants, cookies with filling, candies, and soft drinks, except for nonfried root vegetables, compared to SOC.

Likewise, Durán et al. [38], found that HNS had higher consumption of potatoes (*p* < 0.05), rice (*p* < 0.05), chicken (*p* < 0.05), fish (*p* < 0.01) and milk (*p* < 0.01) than SOC. However, there was significantly lower consumption of bread (*p* < 0.05), vegetables (*p* < 0.05), sausage (*p* < 0.01), pate (*p* < 0.01), beverages (*p* < 0.01) and hot dog (*p* < 0.01) was reported by HNS than SOC. Our results are similar in terms of milk and beverage intake, however, they differ in the consumption of nonfried vegetables and tubers. In turn, Muñoz de Mier et al. [39] indicate that students of health sciences (SHS) at a Spanish university had a significantly higher frequency of consumption of other dairy derivatives (*p* = 0.005) and fast food, precooked food, sauces, salty foods, among others (*p* = 0.048) but a lower frequency of eggs (*p* = 0.046) than SOC. These results are opposite, to some extent, in the case of eggs, to the findings of the present investigation. In contrast, Pérez-Gallardo et al. [40], found no significant difference in the frequency of food consumption between SHS and SOC, except for alcohol consumption, which was significantly (*p* = 0.027) higher in SOC; perhaps this was limited by the small sample size in that study (*N* = 70). In fact, the study by Rizo-Baeza et al. conducted in 184 Spanish SHS corroborates the above, finding no significant differences in diet quality between HNS and nursing students [41]. Thus, in general, other investigators report inconsistencies in the frequency of consumption of healthy and unhealthy foods, but with a tendency toward selection of some healthy foods by HNS and SHS compared to SOC. All the above point to the fact that knowledge of nutrition does not always necessarily translate into a healthy diet in those who have at least one nutrition course in their respective curricula [37].

Despite this, our study shows the greatest number of significant differences between the two groups, with HNS consuming more frequently healthy foods (except for alcoholic beverages) and less frequently unhealthy foods (except for nonfried tubers). These differences could be due, mainly, to the HNS's own knowledge of nutrition [42], the fact that almost three-quarters of these were women, in whom the literature indicates that they have a better nutritional quality than men, and that in this case they do apply although not in their totality [43]. In addition to the above, the results could be favored because in the HNS group there was a considerable percentage of vegetarians. Related to this, several studies, including AHS-2, have shown that vegetarians consume more plant foods such as vegetables, fruits, whole grains, legumes, and nuts or seeds; and less meat, dairy products, eggs, refined cereals, sweets or snacks and sugary beverages [44].

On the other hand, the majority of the students (93.7%) are members of the SDA. This religious organization is known for promoting a healthy lifestyle, including physical activity, vegetarian diet, water consumption, among others, in its members [45]. Likewise, the Adventist lifestyle has been studied by different researchers, resulting in a large body of scientific literature that supports the global influence of SDA, mainly in diet [46]. In addition, all students of this university, regardless of their major, take the course “Health and Physical Culture I and II” in the first year of their degree. In this course, the student is required, as part of their grade, to make behavioral changes in their lifestyle, including their diet. These factors could explain why the sample in general tends to a low frequency of consumption of meats, dairy products, oils and fats, sweets and pastries, and beverages; and a medium-high frequency of intake of cereals, tubers, vegetables, fruits and legumes. This is similar to a semivegetarian or provegetarian diet [44].

### 4.3. Correlation between Type of Career and Dietary Regimen Evaluated

According to the relationship between the type of career and diet evaluated, the result was that there was a significant relationship between these two variables. This is possibly due to the fact that some of the applicants may already be practicing a vegetarian diet beforehand. Therefore, they prefer to opt for a career related to food, such as Human Nutrition. It should be taken into account that a large percentage of the students are Adventists and especially in the HNS there were 72.5% women. In that sense, Adventist students may come predisposed towards a semivegetarian diet that may be finalized when the HNS take the “Vegetarian Diet” course in the 2nd year of the course [47]. Emphasizing that the Professional School of Human Nutrition has within its graduate profile the Vegetarian Nutrition rubric [48]. In addition, it is well known that the practice of a vegetarian diet is more popular among women than among men [49].

### 4.4. Limitations and Strengths

The results of this study should be interpreted within the context of some limitations. First, the small sample and type of population is due to the fact that, in general, analyses comparing BMI or overweight/obesity between vegetarians and nonvegetarians are performed in large, community-based populations [36]. In this regard, it is worth considering that outreach to students was limited by the COVID-19 pandemic, which was first detected in late 2019 [50]. This caused social isolation or quarantine that led to lifestyle changes (such as physical activity, eating habits, among others) in the general population [51], including university students [52]. Second, the anthropometric data (weight and height) were self-referenced, which is subject to potential bias [53] not being properly measured by the appropriate equipment (scale and calibrated measuring rods) and qualified personnel. Other limitations to consider are the inclusion of one university, the type of sampling, which was not representative, and the cross-sectional study design, the latter of which prevents the establishment of cause-effect relationships in the results.

Despite these possible limitations, this research is of particular interest because it is the first study to analyze and classify the diet of Peruvian university students by means of a dietary assessment, namely, an FFQ, given that in other reports the vegetarian and nonvegetarian diet was only self-defined [8]. Finally, this is the first study that compares the frequency of food consumption between HNS and SOC in Peru. In fact, more significant differences were found in both groups compared to other studies [38–40] and consistent results, i.e., in favor of HNS, who had a higher frequency of consumption of healthy foods and lower frequency of consumption of unhealthy foods.

## 5. Conclusions

According to the results, there was no evidence of a significant difference in diet and overweight/obesity between HNS and SOC from a private university in Lima, Peru. However, there were statistically significant differences in the frequency of consumption of some healthy and unhealthy foods between HNS and Peruvian SOC. Due to the limitations of the present study, it is recommended to develop research with a larger population of university students, to replicate it in other universities and with a longitudinal design.

## Figures and Tables

**Table 1:** Characteristics of students according to type of career.

Variable	Human nutrition (*n* = 69)		Other careers^a^ (*n* = 89)		Total (*n* = 158)	
	*n*	%	*n*	%	*n*	%
Age (mean ± SD)	22.2 ± 3.1 years		22.3 ± 4.1 years		22.3 ± 3.7 years	
Sex						
Male	19	27.5	46	51.7	65	41.1 58.9
Female	50	72.5	43	48.3	93	
Place of origin						
Coast	39	56.5	41	46.1	80	50.6
Sierra	20	29.0	30	33.7	50	31.6
Jungle	7	10.1	12	13.5	19	12.0
Foreign	3	4.3	6	6.7	9	5.7
Religion						
Adventist	62	89.9	86	96.6	148	93.7
Others^b^	7	10.1	3	3.4	10	6.3
Faculty						
FBS	69	100	8	9.0	8	5.1
FHSE			8	9.0	8	5.1
FHS			39	43.8	108	68.4
FEA			20	22.5	20	12.7
FT			14	15.7	14	8.9
Year of study						
1st	6	8.7	7	7.9	13	8.2
2nd	10	14.5	16	18.0	26	16.5
3rd	18	26.1	29	32.6	47	29.7
4th	26	37.7	23	25.8	49	31.0
5th	9	13.0	12	13.5	21	13.3
6th	0	0.0	1	1.1	1	0.6
7th	0	0.0	1	1.1	1	0.6
Presence of disease						
Yes	7	10.1	12	13.5	19	12.0
No	62	89.9	77	86.5	139	88.0
Use of medications						
Yes	5	7.2	5	5.6	10	6.3
No	64	92.8	84	94.4	148	93.7
Use of supplements						
Yes	5	7.2	3	3.4	8	5.1
No	64	92.8	86	96.6	150	94.9

^a^It included administration; architecture; communication sciences; accounting; education; nursing; environmental, civil, food and systems engineering; medicine; psychology and theology. ^b^It included Catholic, Evangelical, Mormon, and no religion. FBS, Faculty of Business Sciences; FHSE, Faculty of Human Sciences and Education; FHS, Faculty of Health Sciences; FEA, Faculty of Engineering and Architecture; FT, Faculty of Theology.

**Table 2:** Comparison of overweight/obesity between career type and self-reported dietary regimens.

Variable	Men (*n* = 65)		Women (*n* = 93)		*X* ^2^^*∗*^	*p* value
	*n*	%	*n*	%		
Diagnosis by BMI					6.191	**0.013** ^*∗∗*^
Thinness/normal	40	61.5	74	79.6		
Overweight/obesity	25	38.5	19	20.4		

	Human nutrition (*n* = 69)		Other careers^a^ (*n* = 89)			
	*n*	%	*n*	%		

Diagnosis by BMI					0.079	0.779
Thinness/normal	49	71.0	65	73.0		
Overweight/obesity	20	29.0	24	27.0		
Dietary regimen					0.238	0.625
Nonvegetarians	46	66.7	56	62.9		
Vegetarians	23	33.3	33	37.1		

	Nonvegetarians (*n* = 102)		Vegetarians (*n* = 56)			
	*n*	%	*n*	%		

Diagnosis by BMI					0.350	0.554
Thinness/normal	72	70.6	42	75.0		
Overweight/obesity	30	29.4	14	25.0		

^*∗*^Chi-square test. ^*∗∗*^p significant. ^a^It included administration; architecture; communication sciences; accounting; education; nursing; environmental, civil, food and systems engineering; medicine; psychology and theology. BMI, body mass index.

**Table 3:** Comparison of the frequency of food consumption by type of career.

Food groups	Foods	Human nutrition (*n* = 69)	Other careers^a^ (*n* = 89)	*U* ^*∗*^	*p* value^*∗∗*^
		Median	Median		
Tubers	Potato, sweet potato, cassava, olluco, chuño^1^	71.3	85.9	2505.0	**0.039**
	French fries or fried sweet potato^1^	65.1	90.6	2080.0	**<0.001**
Vegetables	Yellow or orange color: Carrot, pumpkin, squash^1^	88.7	72.4	2436.0	**0.020**
	Purple color: beet, eggplant, purple cabbage^1^	87.4	73.4	2528.5	**0.049**
Fruits	Citrus: orange, tangerine, kiwi, strawberry^1^	88.1	72.8	2476.5	**0.029**
Meats	Eggs^2^	91.5	70.2	2245.5	**0.002**
Dairy and dairy products	Whole milk^1^	89.5	71.7	2378.5	**0.013**
	Yogurt^1^	89.1	72.1	2409.5	**0.017**
	Custard, pudding, flan^1^	72.2	85.2	2565.0	**0.048**
Soy and soy products	Tofu or soy cheese^1^	91.2	70.4	2261.0	**0.003**
Oils and fats	Olive oil^1^	91.5	70.2	2245.0	**0.003**
	Sunflower, corn, soybean oil^1^	96.9	66.0	1870.0	**<0.001**
	Flour fritters^1^	70.7	86.4	2460.0	**0.027**
Sweet and bakery products	Chocolate cookies^1^	71.7	85.6	2531.0	**0.050**
	Pies, croissants^1^	71.0	86.1	2480.5	**0.030**
	Cookies with filling^1^	70.5	86.5	2448.5	**0.024**
	Candies, mints^1^	68.6	87.9	2319.0	**0.006**
Beverages	Soft drinks^1^	70.1	86.8	2425.0	**0.016**
	Alcoholic beverages^1^	84.3	75.8	2742.5	**0.037**

^*∗*^Mann-Whitney U test. ^*∗∗*^only significant results. ^a^It included administration; architecture; communication sciences; accounting; education; nursing; environmental, civil, food and systems engineering; medicine; psychology and theology. ^1^0 = 0 times/week, 1 = 0.25 times/week, 2 = 0.5 times/week, 3 = 1.5 times/week, 4 = 3.5 times/week, 5 = 5.5 times/week and 6 = 7 times/week. SD, standard deviation.

**Table 4:** Relationship between the type of career and type of diet evaluated.

Variable	Human nutrition (*n* = 69)		Other careers^a^ (*n* = 89)		*X* ^2^^*∗*^	*p* value
	*n*	%	*n*	%		
Dietary regimen					5.378	0.020^*∗∗*^
Nonvegetarians	56	81.2%	83	93.3%		
Vegetarians	13	18.8%	6	6.7%		

^*∗*^Chi-square test. ^*∗∗*^p significant. ^a^It included administration; architecture; communication sciences; accounting; education; nursing; environmental, civil, food and systems engineering; medicine; psychology and theology.

## Data Availability

The datasets used and analyzed during the present study are available from the corresponding author on reasonable request.
